# Insights Into the Pathologic Roles and Regulation of Eukaryotic Elongation Factor-2 Kinase

**DOI:** 10.3389/fmolb.2021.727863

**Published:** 2021-08-31

**Authors:** Darby J. Ballard, Hao-Yun Peng, Jugal Kishore Das, Anil Kumar, Liqing Wang, Yijie Ren, Xiaofang Xiong, Xingcong Ren, Jin-Ming Yang, Jianxun Song

**Affiliations:** ^1^ Department of Microbial Pathogenesis and Immunology, Texas A&M University Health Science Center, Bryan, TX, United States; ^2^ Department of Biochemistry and Biophysics, Texas A&M University, College Station, TX, United States; ^3^ Department of Toxicology and Cancer Biology, University of Kentucky College of Medicine, Lexington, KY, United States

**Keywords:** cancer, drug development, mTORC1, AMPK, eEF2K, immunometabolism, signaling pathways, protein kinase

## Abstract

Eukaryotic Elongation Factor-2 Kinase (eEF2K) acts as a negative regulator of protein synthesis, translation, and cell growth. As a structurally unique member of the alpha-kinase family, eEF2K is essential to cell survival under stressful conditions, as it contributes to both cell viability and proliferation. Known as the modulator of the global rate of protein translation, eEF2K inhibits eEF2 (eukaryotic Elongation Factor 2) and decreases translation elongation when active. eEF2K is regulated by various mechanisms, including phosphorylation through residues and autophosphorylation. Specifically, this protein kinase is downregulated through the phosphorylation of multiple sites *via* mTOR signaling and upregulated *via* the AMPK pathway. eEF2K plays important roles in numerous biological systems, including neurology, cardiology, myology, and immunology. This review provides further insights into the current roles of eEF2K and its potential to be explored as a therapeutic target for drug development.

## Introduction

Eukaryotic cells tightly regulate protein synthesis, a biological process critical for cell survival, proliferation, and function. Eukaryotic Elongation Factor 2 Kinase (eEF2K) is a crucial regulator of protein synthesis *via* inhibiting protein translation to conserve the limited energy of the cell (Ryazanov et al., 1997). Through the lens of cancer therapeutics, eEF2K is a valid and prominent target, as primary research on focused on the protein kinase’s role in cancer. It was found that overexpression of eEF2K permits increased tumor-cell survival in many types of cancer, including breast, brain, pancreatic, and lung cancer (Karakas and Ozpolat, 2020; Xiao et al., 2020). Despite its prevalence in cancer development, eEF2K is also vital in the immune response during infection as it plays a critical role in the metabolic regulation of different immune cells. The importance of eEF2K in neurobiology as well as in myology are also active areas of research. Here, we review the recent advances in understanding the pathophysiological roles of eEF2K and its regulation. We also discuss how alterations in metabolism regulated by eEF2K relate to immunity in different pathologies.

## eEF2K Structure and Function

Protein synthesis is a controlled chemical reaction that consumes an estimated 30–50% of the cell’s total energy (Browne et al., 2004). This process occurs through three principal steps: initiation, elongation, and termination, and results in the formation of a polypeptide (Griffiths, 2000). The elongation process requires substantial metabolic energy and is regulated by multiple elongation factors (Browne and Proud, 2002). One of these elongation factor regulators, eEF2K, acts by controlling elongation through phosphorylation of eukaryotic elongation factor 2 (eEF2), reducing mRNA translation rates in cells (Hizli et al., 2013).

eEF2K is a member of the alpha-kinase family, a distinct class of kinases that have tendencies to phosphorylate molecules in alpha-helices that show little sequence homology with the conventional kinase protein family (Ryazanov et al., 1997). The alpha-kinase family represents less than ten percent of the characterized kinase proteins known to date, and other members of this class include alpha-kinase 1 (lymphocyte alpha-kinase, LAK, or ALPK1) and alpha-kinase 3 (muscle alpha-kinase, MAK, or ALPK3) (Middelbeek et al., 2010). eEF2K is dependent upon both the Ca^2+^ ions and calmodulin (CaM) for its kinase activity, differentiating it from other members of the alpha-kinase family that do not depend upon Ca^2+^/CaM-binding (Nairn and Palfrey, 1987; Ryazanov et al., 1988). Despite this, eEF2K shares no homology with other Ca^2+^/CaM-dependent protein kinases. Furthermore, eEF2K phosphorylates threonine, whereas other Ca^2+^/CaM kinases target serine residues (Middelbeek et al., 2010).

**FIGURE 1:**
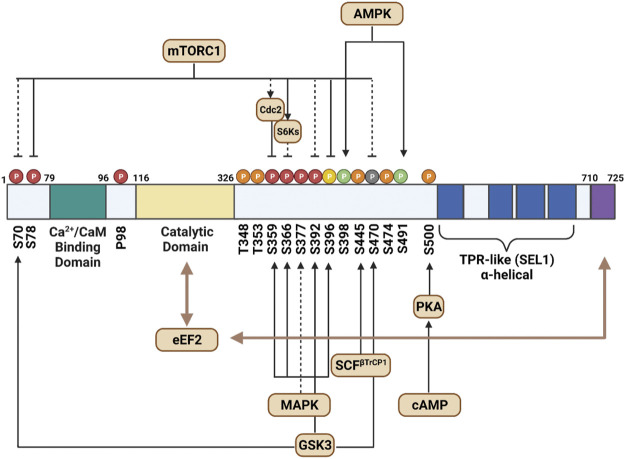
This schematic outlines the structure of eEF2K and the primary functional features of the molecule. It additionally depicts the direct (solid lines) and indirect (dashed lines) signaling pathways that moderate its activity. The end of the C-terminal, shown in purple, assists the catalytic domain, characterized in yellow, to phosphorylate eEF2. The phosphorylated residues shown in green indicate sites that activate eEF2K activity, and residues in red depict inhibited eEF2K sites. The auto-phosphorylated residues that trigger eEF2K are shown in orange. Sites shown in yellow represent residues that indirectly affect the phosphorylated sites near N-terminus, and gray residues are those that have known phosphorylation activity but do not affect eEF2K activity.

eEF2K consists of four main components: the calmodulin-binding domain, the catalytic domain, a regulatory loop, and a TPR-like alpha-helical region (Figure 1). The N-terminal calmodulin-binding domain is the site where CaM attaches and triggers eEF2K’s activation cascade (Pigott et al., 2012). The regulatory loop (residue 326–480) contains the most phosphorylated sites and is fundamental to the kinase’s function. It is believed that the C-terminal region neighbors a series of alpha-helical (SEL1) repeats, but a more detailed model is needed for confirmation (Mittl and Schneider-Brachert, 2007). The function of the center of SEL1 repeats is currently uncharacterized but is thought to play a role in folding and protein-protein interaction. The extreme C-terminal of eEF2K is critical for eEF2 phosphorylation, but there is no primary binding site for eEF2 (Will et al., 2016).

## eEF2K Regulation

The activity of eEF2K is regulated at several levels and is preceded by a sequential two-step mechanism. First, Ca^2+^ and CaM bind to the Ca^2+^/CaM-binding domain of eEF2K, activating its *a*-kinase catalytic domain. Activation of the catalytic domain is followed by rapid autophosphorylation of Thr-348, the second component of the activation mechanism. Autophosphorylation of Thr-348 triggers a conformational change of the kinase domain, increasing the activity of eEF2K (Tavares et al., 2012; Tavares et al., 2014). Although there are four other major Ca^2+^/CaM-stimulated autophosphorylation sites in eEF2K (Thr-353, Ser-445, Ser-474, and Ser-500), Thr-348 is the first site to be autophosphorylated and is critical for eEF2K activation (Abramczyk et al., 2011; Tavares et al., 2014). The level of eEF2K protein can also be self-regulated. A study conducted by Wang et al. showed that eEF2K stability was self-regulated, as its degradation required phosphorylation at Thr-348, which becomes the stabilized mutant T348A once degraded (Wang et al., 2015).

eEF2K is a complexly regulated protein kinase, as specific residue-phosphorylation sites can either render it active or inactive. These phosphorylation events closely correspond to the nutrient status of cells, a hallmark of cellular metabolism and health (Table 1). In the S6K-mediated pathway, ribosomal protein S6 kinase beta-1 (RPS6KB1) or p70 S6 kinase (p70S6K), which phosphorylates the S6 ribosomal protein to induce protein synthesis, can also phosphorylate eEF2K on Ser-366, rendering it inactive (Wang et al., 2001). Because multiple growth factors and MAPK signaling proteins can activate the mTOR pathway, mTOR and p70S6K serve as critical signaling proteins linking eEF2K activity and cell growth to the metabolic state of the cell (Sengupta et al., 2010).

**TABLE 1:** eEF2K phosphorylated sites with their effect and mechanism.

Residue	Mechanism	Effect	References
Ser-70	mTORC1, GSK3 *in vivo*, insulin	Inhibitory	Wang et al. (2014)
Ser-78	mTORC1	Inhibitory	Browne and Proud (2004)
Pro-98	Hydroxylation	Inhibitory	Moore et al. (2015)
Tyr-348	Activation of the catalytic domain	Autophosphorylation	Abramczyk et al. (2011); Tavares et al. (2012); Tavares et al. (2014)
Tyr-353	-	Autophosphorylation	Tavares et al. (2012); Tavares et al. (2014)
Ser-359	mTORC1, ERK, P38δ, cdc2-cyclinB complex, SAPK4, agonists, insulin	Inhibitory	Knebel et al. (2001); Smith and Proud (2008); Wang et al. (2014); Wang et al. (2018)
Ser-366	mTORC1, P70S6K, p90^RSK1^	Inhibitory	Wang et al. (2001); Wang et al. (2014)
Ser-377	Agonists, MAPKAP-K2/K3 substrate	Inhibitory	Knebel et al. (2002); Wang et al. (2018)
Ser-392	mTORC1/mTORC2, insulin, GSK3 *in vivo*	Inhibitory	Wang et al. (2014)
Ser-396	P38 MAPK *in vitro*, mTORC1	Indirectly affects the phosphorylated sites	Knebel et al. (2002); Wang et al. (2014)
Ser-398	AMPK	Activation	Browne and Proud (2004); Hardie (2011)
Ser-445	SCF ^βTrCP1^ ubiquitin ligase	Autophosphorylation	Kruiswijk et al. (2012); Pyr Dit Ruys et al. (2012); Tavares et al. (2012); Tavares et al. (2014)
Ser-470	GSK3 *in vivo*, Insulin, mTORC1	-	Wang et al. (2014)
Ser-474		Autophosphorylation	Tavares et al. (2012); Tavares et al. (2014)
Ser-491	AMPK	Activation	Tong et al. (2020)
Ser-500	PKA substrate	Activation/Autophosphorylation	Diggle et al. (2001); Tavares et al. (2012); Tavares et al. (2014)

**FIGURE 2:**
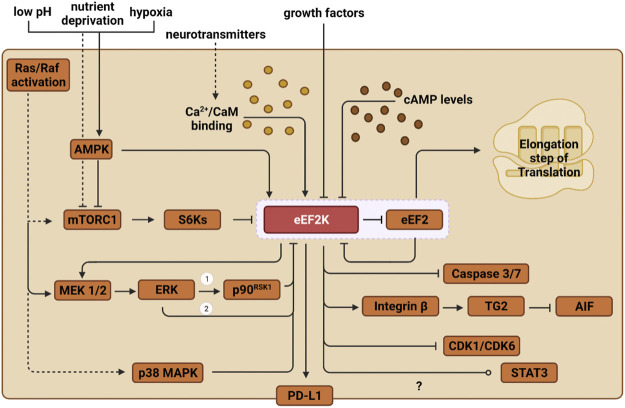
Outlined are the various pathways through which eEF2K is regulated and how it ultimately impacts protein synthesis. Solid lines depict direct pathways, and indirect pathways are shown with dashed lines. eEF2 is the only substrate for eEF2K, shown in the red box. ERK regulates eEF2K in two ways: 1) direct inhibition on eEF2K and 2) the induction of p90^RSK1^. The regulation of STAT3 by eEF2K is under debate, but current evidence shows eEF2K inhibits STAT3 in lung cancer and activates it in liver cancer.

Because the activity of eEF2K directly affects the rate of protein translation, it is unsurprising that eEF2K activation is sensitive to the cell’s metabolic state. Therefore, in addition to regulation by Ca^2+^/CaM, eEF2K is also regulated by phosphorylation *via* other kinases. The two primary mechanisms that regulate eEF2K phosphorylation are the mTORC1 and AMPK pathways. During nutrient deprivation, AMPK directly phosphorylates Ser-398 on eEF2K, activating the residue and upregulating eEF2K (Figure 2) (Hardie, 2011). Genotoxic stress-induced by DNA intercalating agents such as doxorubicin have also been shown to activate eEF2K through the AMPK-mediated phosphorylation on Ser-398 (Kruiswijk et al., 2012).

Ser-78 is uniquely positioned in eEF2K as it sits next to the Ca^2+^/CaM-binding domain (Browne and Proud, 2004). Studies have shown that mTORC1 phosphorylates Ser-78 in a rapamycin-sensitive manner and effectively prevents Ca^2+^/CaM binding when introduced to insulin. Lack of binding prevents eEF2K activation and allows for an increase in translation elongation activity from eEF2. Ser-498 acts similarly through increased cAMP levels to ultimately downregulate eEF2K activity *via* SAPK4 on Ser-359 (Knebel et al., 2001; Wang et al., 2001; Will et al., 2016).

Several growth factors have been shown to affect the activity of eEF2K. For example, insulin-like growth factor 1 (IGF-1) and epidermal growth factor (EGF) both induce the phosphorylation of Ser-359, rendering eEF2K inactive (Chen et al., 2008). eEF2K is controlled by pH, being acutely activated in cells during acidosis (Xie et al., 2015). Interestingly, eEF2K is also regulated by feedback inhibition *via* eEF2 activity (Mccamphill et al., 2015). Cyclin A–cyclin-dependent kinase 2 (Cdk2) can phosphorylate eEF2 on Ser-595, directly regulating Thr-56 phosphorylation on eEF2K (Hizli et al., 2013).

eEF2K can be regulated by post-translational modifications apart from phosphorylation. For example, eEF2K activity is inhibited when hydroxylated on proline residue 98, preventing its binding to CaM (Moore et al., 2015). This regulation is sensitive to oxygen levels, as proline hydroxylation is impaired during hypoxia and hinders protein synthesis. Subsequently, this mechanism allows cells to adapt to reduced oxygen availability. eEF2K is a target of SCF (Skp, Cullin, F-box–containing) βTrCP (β-transducin repeat-containing protein) E3 ubiquitin ligase *via* Ser-441 and Ser-445, which promotes its degradation and subsequent downregulation (Kruiswijk et al., 2012; Meloche and Roux, 2012). eEF2K can additionally complex with heat shock protein 90 (Hsp90), and disruption of the eEF2K-Hsp90 combination causes its ubiquitination and eventual degradation (Arora et al., 2005).

## eEF2K Activity and Cell Signaling in Neurology

eEF2K function is crucial to the nervous system and an imperative component of certain neuro-pathologies (Park et al., 2008). Suppression of *N*-methyl-*D*-aspartate receptor (NMDAR) by the antidepressant drug, ketamine, reduces the phosphorylation of eEF2 *via* activation of eEF2K and is the principal molecular device for controlling synaptic plasticity and memory function (Li and Tsien, 2009; Autry et al., 2011; Duman et al., 2012; Monteggia et al., 2013; Adaikkan et al., 2018). The receptor for glutamate, an excitatory amino acid transmitter, regulates eEF2K phosphorylation through the concentration of calcium ions in a timely and dose manner, which controls synaptic plasticity (Barrera et al., 2008; Costa-Mattioli et al., 2009). eEF2K-defective mice with reduced kinase activity demonstrated impaired cortical-dependent associative, but not incidental, taste learning (Gildish et al., 2012).

Furthermore, recent studies have shown that eEF2K regulates protein translation in dendrites (Heise et al., 2014). Through these experiments, it was found that activation of eEF2K functioned as a biochemical sensor in dendrites where spontaneous neurotransmitter release (i.e., miniature neurotransmission) strongly promoted the phosphorylation and inactivation of eEF2 in cultured hippocampal neurons (Sutton et al., 2007). Using both pharmacological and genetic manipulation, the synthesis of proteins related to neuronal microtubule processes and intracellular trafficking such as Nsf and Map2 were found to be downstream of and regulated by eEF2K, supporting its role in neuron cytoskeleton architecture (Kenney et al., 2016). These observations suggest that changes in protein synthesis due to eEF2K inhibition are unlikely to be attributed to alterations in mRNA expression.

eEF2K function is also crucial in vascular biology in the brain (Kameshima et al., 2016) and is associated with the proliferation of rat glial cells (Bagaglio and Hait, 1994). Micro-RNAs (miRs) function in RNA silencing and post-transcriptional regulation of gene expression. Neurons secrete exosomes containing miR-132, which are internalized to endothelial cells where they can affect eEF2K expression (Xu et al., 2017). This expression mediates the activity of miR-132 on adherens junction protein Vascular Endothelial-cadherin (VE-cadherin, also known as Cdh5) expression and brain-vascular integrity (Xu et al., 2017). Synaptic plasticity and memory are essential processes in learning that require efficient protein synthesis regulated by mTORC1 (Im et al., 2009; Kenney et al., 2015). eEF2K activity directly affects this plasticity in motor neurons, which show decreased eEF2 phosphorylation as a critical downstream effector of mTOR (Mccamphill et al., 2017).

Because eEF2K impacts synaptic plasticity, studies have been conducted to investigate the ties between eEF2K and neurodegenerative diseases, including Alzheimer’s disease (AD), Parkinson’s disease, and epilepsy (Jan et al., 2018; Beckelman et al., 2019). Increased expression of eEF2K and decreased expression of eEF2 have been observed in the cortex and hippocampus of AD patients, alluding to the significance of eEF2K’s role in brain function and memory (Li et al., 2005; Jan et al., 2017). Ablation of eEF2K prevents amyloid-β 42 (Aβ42) oligomers from impairing synaptic plasticity, the hallmark feature in AD (Jan et al., 2017). Furthermore, the accumulation of aggregated alpha-synuclein (AS) protein in the brain, which triggers synaptic dysfunction and oxidative stress, is implicated in Parkinson’s disease (Hsu et al., 2000; Jan et al., 2018). Inhibiting eEF2K reduces these harmful AS protein aggregates by decreasing ROS and oxidative stress levels (Jan et al., 2018). The imbalance of inhibitory and excitatory synaptic transmission has been demonstrated in the pathogenesis of epilepsy (Heise et al., 2017). A recent study indicated that eEF2K activity negatively impacts the signaling at the GABAergic synapse (Heise et al., 2017). eEF2K-deficient mice display a more robust GABAergic signaling and exhibit a rescue effect on epilepsy symptoms, suggesting that pharmacological or genetic inhibition of eEF2K could potentially counteract disease phenotypes. (Heise et al., 2017).

## eEF2K in Cancer

Recent research has shown that eEF2K is associated with tumor survival, proliferation, migration, and invasion (Xie et al., 2014; Faller et al., 2015; Zhu et al., 2017; Ng et al., 2019; Liu et al., 2020; Tong et al., 2020; Wu et al., 2020; Xiao et al., 2020; Erdogan et al., 2021; Ju et al., 2021). The proliferation of cancer cells requires a large amount of energy to grow due to the high demands of protein synthesis and the generation of building blocks. These biologic demands lead to the creation of the harsh and acidic tumor microenvironment, lacking nutrients, energy deficiency, and insufficient oxygen levels. Cancer cells invoke cytoprotective responses that trigger resistance mechanisms to assist poorly vascularized tumors in surviving this environment (Xiao et al., 2020). For instance, eEF2K is upregulated to regulate a high rate of protein synthesis (Liu et al., 2020; Erdogan et al., 2021; Temme and Asquith, 2021). A study conducted by Ju indicated that the inhibition of eEF2K, with the glutamine starvation, induces cyclin-dependent kinase 1 (CDK1) and 6 (CDK6), which then suppress the growth of triple-negative breast cancer (Ju et al., 2021).

AMPK activation promotes tumor cell survival by inhibiting the interaction between eEF2K and mitogen-activated protein kinase (MEK1/2) under nutrient deprivation. In contrast, in the presence of nutrients, eEF2K provides a positive feedback loop *via* MEK1/2-ERK1/2-ribosomal protein S6 kinase signaling (Tong et al., 2020). As for prostate and lung cancer cells, a study from Wu has shown that the absence of eEF2K has reduced the expression of PD-L1 protein, an immune checkpoint protein, to help cancer cells to escape from immune cells (Wu et al., 2020). In esophageal squamous cell carcinoma (ESCC), eEF2k express higher than non-tumor tissues, and ablation of eEF2K correlated with slower migration and proliferation rate (Zhu et al., 2017).

However, contradictory studies have surfaced at which point eEF2K disrupts the growth of certain cancers, including colon tumors, intestinal tumors, and lung tumors (Xie et al., 2014; Faller et al., 2015; Ng et al., 2019; Xiao et al., 2020). eEF2K inhibits the aerobic glycolysis of lung cancer cells by the blockage of pyruvate kinase M2 isoform (PKM2) dimerization and inactivation of STAT3, leading to the impairment of tumor growth (Xiao et al., 2020). Surprisingly, these effects of eEF2K are independent of its function-inhibiting protein synthesis (Xiao et al., 2020). The expression of eEF2K is downregulated in colon cancer patients, which is associated with worse overall survival (Ng et al., 2019). This suggests the dual role of eEF2K in multiple cancer cell lines, and future investigation is required.

## eEF2K Activity in Immune Cells and Pathologies

Cells of the immune system receive fluctuating signals to synthesize proteins at different rates in response to various physiological stresses, including temperature changes, ultraviolet (UV) irradiation, nutrient limitation, oxidative stress, hypoxia, and exposure to various drugs or toxins and infections (Holcik and Sonenberg, 2005; Mace et al., 2011; Weill et al., 2011; Buck et al., 2015).

B-cell-activating factor (BAFF) is a cytokine that belongs to the TNF superfamily and is produced by monocytes, dendritic cells, B cells, and some T cells (Mackay et al., 2007). Interestingly, the expression of eEF2K was significantly decreased in the BAFF-stimulated cells as compared to untreated control cells (Saito et al., 2008). All the three B cell leukemia cell lines, Sup-B15, Reh, and SEM, showed a decrease in the phosphorylation of the inactivating eEF2K residue Ser-366 when treated with proteasome deubiquitinase inhibitor VLX1570 and/or L-asp (Mazurkiewicz et al., 2017). Using a doxycycline (dox)- inducible system, depletion of ribosomal protein (RP) mRNA induced activation of eEF2K and eEF2 phosphorylation in human-human erythroleukemia K562C cells, resulting in inhibition of protein elongation (Gismondi et al., 2014). In a different system using an inactive mutant of the PeBoW complex, a protein complex involved in coordinating ribosome biogenesis with cell cycle progression, both eEF2K activity and expression were reduced *via* signaling downstream of mTOR (Holzel et al., 2005; Liu et al., 2014). These results indicate the significance of eEF2K signaling in regulating ribosome biosynthesis and function, which directly impacts cell viability.

The inflammatory response is an essential biological process that occurs during tissue injury or destruction and has a vital role in preventing the tissue from further damage. Activation of the inflammatory response may or may not be accompanied by infection. Several studies have shown the importance of eEF2K signaling pathways in inflammation and inflammatory processes. For example, eEF2K has been shown to mediate reactive oxygen species (ROS)-dependent vascular inflammation and is partly responsible for hypertension *via* propagating vascular hypertrophy and endothelial dysfunction in rats (Usui et al., 2013). eEF2K knockdown significantly inhibited monocyte adhesion to human umbilical vein endothelial cells (HUVECs) (Usui et al., 2013). One of the well-characterized inflammatory signals is the cytokine TNF-α, which has been shown to be induced by ROS in endothelial cells and is a significant contributor to inflammation (Chen et al., 2008; Peng et al., 2021). Knockdown of eEF2K significantly reduces TNF-α induced ROS production in HUVECs (Usui et al., 2013).

Viral infection can dramatically disrupt normal protein synthesis as viruses rely on the translation machinery of their host cells to produce polypeptides required for viral replication (Walsh and Mohr, 2011). Several studies have shown that pathogens, including viral and bacterial agents, disrupt the key effectors of the protein translation machinery and are essential in host defense. For example, infection of human gastric adenocarcinoma (AGS) cells with wild-type or the cytotoxin-associated gene A (CagA) and virB7 mutants of *H. pylori* suppressed eEF2 phosphorylation at Thr-56 on eEF2K at 1 to 3 hours post-infection (Sokolova et al., 2014). CagA is an *H. pylori* virulence factor that is used to inject CagA into a target cell upon *H. pylori* attachment. The virB7 gene is among seven genes essential for CagA translocation into host cells (Fischer et al., 2001). Coxsackievirus B3 (CVB3) infection of cardiomyocytes causes myocarditis (Cooper, 2009). It was reported that the natural compound emodin inhibits CVB3 viral replication by suppressing viral translation elongation by activating eEF2K (Zhang et al., 2016).

Transcriptional profiling of the lymph nodes, blood, and colon samples from simian immunodeficiency virus (SIM) infected African green monkeys (AGM), and Asian pigtailed macaques (PT) showed a shift toward cellular stress pathways and Th1 profiles, with sustained and robust type I and II interferon responses (Lederer et al., 2009). In this study, eEF2K expression was dramatically increased in the lymph nodes extracted from non-pathogenic PT animals 45 days after SIV infection. Interestingly, eEF2K expression was also significantly increased in the natural host AGM animals as early as 10 days after infection. This upregulation was accompanied by CD4^+^ T cell depletion in multiple anatomic compartments in the PT animals.

eEF2K activity regulates protein synthesis of key proinflammatory cytokines such as TNF-alpha during liver disease. For example, the MKK3/6-p38γ/δ pathway was found to mediate inhibitory phosphorylation of eEF2K, which in turn promoted eEF2 activation (dephosphorylation) and subsequent TNF-α elongation during LPS induced liver damage (Gonzalez-Teran et al., 2013). Pathologies of the kidney, manifested by HIV infection, can be characterized by a proliferative phenotype in glomerular and tubular lesions (Rosenberg et al., 2015). Tg26 HIV transgenic mice showed an increase in mTOR activity in renal tissues accompanied by the rise in eEF2K phosphorylation (Kumar et al., 2010). Infection by bacterial pathogens such as *Streptococcus pneumoniae* has been shown to affect the host response *via* protein translation. For example, eEF2K knock-out mice showed significantly reduced bacterial clearance in lung tissue compared to wild-type (WT) mice infected with *Streptococcus pneumoniae* after 24 h (Bewley et al., 2011). In a study using whole-genome arrays, gene expression profiles from circulating peripheral blood leukocytes were compiled from patients with melioidosis and tuberculosis. It was observed that eEF2K expression was significantly downregulated in patients with tuberculosis but not in patients with melioidosis, which involved three major gene sets, including glypican networks, TGF-β receptor signaling, regulation of SMAD2/3, and IFN-γ pathways (Koh et al., 2013).

The study of the role of eEF2K in T cells and its pathologies is just beginning. In T cells, eEF2K plays an essential role in cell division. For example, in response to activated protein kinase A (PKA), immortalized Jurkat cells downregulate the expression of Cyclin D3. Cyclin D3 is required for cell division and acts by decreasing the rate of translation elongation caused by increased eEF2K activity and subsequent eEF2 phosphorylation (Gutzkow et al., 2003). In a study to examine how TGF-β contributes to nephropathy, activation of eEF2 *via* inactivation of eEF2K by p90^Rsk^ (Das et al., 2010) was observed. This inactivation coincided with mesangial cell hypertrophy.

Recent studies have demonstrated the significance of dysregulation of protein translation in autoimmune disorders. For example, NOD mice model the autoimmune pathology of type 1 diabetes (T1D), where the earliest signs of pathology in the pancreas occur at 4 weeks of age (Thomas and Kay, 2000). Transcriptome analysis of leukocytes extracted from the spleen of four-week-old mice showed abnormal expression of several metabolic pathway genes, including eEF2K, compared to transcript expression in spleen leukocytes from two-week-old mice (Wu et al., 2012). Further involvement of eEF2K in autoimmune disorders was revealed in a study that detected anti-eEF2K antibodies from sera of systemic lupus erythematosus patients (Arora et al., 2002). These studies highlight the advances in our understanding of the role of eEF2K in immunology.

## Additional Pathologies Involved in eEF2K Regulation

Various signaling pathways are involved in the regulation of muscle tissue (Fujita et al., 2007). A Ca^2+^–calmodulin–eEF2K–eEF2 signaling cascade, independent of AMPK activity, was shown to contribute to suppressing skeletal muscle protein synthesis during contractions (Rose et al., 2009). One study found that eEF2K protein localized to cardiomyocytes was significantly higher in hypertrophied left ventricle tissue from spontaneously hypertensive rats than normal left ventricle tissue from normal Wistar Kyoto rats (Kameshima et al., 2016). eEF2K was found to partly mediate monocrotaline-induced pulmonary arterial hypertension *via* stimulation of vascular structural remodeling. It is currently thought that this mechanism occurs through the NADPH oxidase-1/ROS/matrix metalloproteinase-2 pathway, but further evidence is needed to validate this finding (Kameshima et al., 2015). eEF2K activity also affects the proliferation and migration of smooth muscle cells of the mesenteric artery of rats (Usui et al., 2015). These studies and others support eEF2K as a novel molecular target for the prevention and treatment of essential hypertension.

Muscular hypertrophy is a characteristic of multiple debilitating pathologies such as cardiomyopathy, amyloidosis, sarcoidosis, debrancher enzyme deficiency, hypothyroid myopathy, and Duchenne’s and Becker’s dystrophy (Kang et al., 2016; Cariou et al., 2017; Wu et al., 2017). Several studies found that the deregulation of protein synthesis *via* eEF2K is implicated in these pathologies. For example, the C2C12 mouse skeletal myoblast cell line presented significantly decreased eEF2 Thr-56 phosphorylation after both static and cyclic stretching (Nakai et al., 2010). Unlike hepatocytes, murine myoblast primary cultures were sensitive to rapamycin treatment that regulates eEF2 phosphorylation (Mieulet et al., 2007). This study showed that, unlike previous reports, eEF2K activity is not directly affected by S6K signaling. The activity of eEF2K has also been reported to influence the development of atherosclerosis. It was observed that mice receiving a bone marrow transplant from eEF2K-expressing mice showed a significant decrease in atherosclerotic plaque formation *via* decreased M1-skewed macrophage secretion of tumor necrosis factor (TNF) (Zhang et al., 2014).

Since protein synthesis occurs in all cell types, it is not surprising that eEF2K plays a role in various biological processes, including the eye and stem cells. A recent study by Olivares et al. established a mouse model for age-related macular degeneration (AMD) in which miR-883, miR-466, and miR-345, micro-RNAs that all target eEF2 were differentially expressed at various time points during retinal development as compared to the retinas from normal C57BL6/J mice (Olivares et al., 2017). Moreover, the expression of eEF2K is both growth factor- and cell line-dependent. Interestingly, eEF2K shows opposing functions in stem cells. One study showed that eEF2K knock-out mice were protected from hematopoietic syndrome yet hypersensitive to gastrointestinal syndrome (Liao et al., 2016). eEF2K also functions to maintain germline quality *via* regulating apoptosis by controlling translation of the anti-apoptotic proteins XIAP and c-FLIPL in oocytes from *C. elegans* (Chu et al., 2014).

## Clinical Applications and Drug Developments

The physiological role eEF2K plays in developing and progressing several diseases, especially cancers, has led researchers to design therapeutics that target the protein kinase. eEF2K is highly expressed and regulates apoptosis, cell survival, autophagy in multiple cancer cells. Importantly, eEF2K is not required for mammalian viability or health under normal conditions (Wu et al., 2020). This leads to a pivotal role in drug development, and eEF2K is mainly being explored as a therapeutic target for numerous diseases, including cancers, neurodegenerative diseases, and cardiovascular diseases (Fujita et al., 2007; Beckelman et al., 2019; Temme and Asquith, 2021). Recent research progress on eEF2K for drug development arises from generating eEF2K inhibitors (Table 2) and further starting several treatments, including PROTAC (Proteolysis target chimeric) technology and combination therapies (Zhu et al., 2017; Liu et al., 2020). eEF2K inhibitors for cancer therapeutics take advantage of the molecule’s signaling and regulatory pathways. Since the activation of eEF2K is related to the levels of calcium ions and AMP/ATP levels, some inhibitors, including NH125, are designed as ATP competitive inhibitors (Arora et al., 2003; Arora et al., 2004; Lockman et al., 2010; Zhang et al., 2011; Zhu et al., 2017; Xie et al., 2018).

**TABLE 2:** The inhibitors/activators targeting eEF2K.

Name of compound	Structure	Principle	Type of pathologies	References
A484954 (7-amino-1-cyclopropyl-3-ethyl-2,4-dioxo-1,2,3,4-tetrahydropyrido [2,3-days] pyrimidine-6-carboxamide)	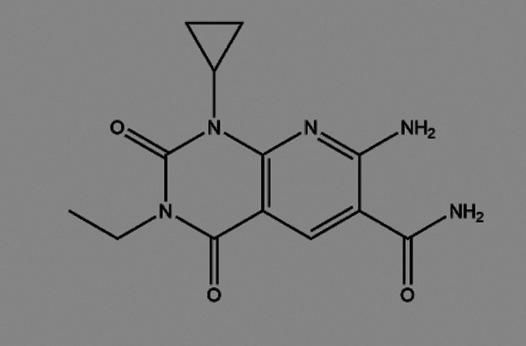	ATP competitive inhibitor, but CaM-independent Demonstrates almost no cytotoxicity	Hypertension	Kodama et al. (2018); Xie et al. (2018); Kodama et al. (2019)
			Atherosclerosis	
			Breast cancer	
			Lung cancer	
			TNBC	
Rottlerin (5,7-dihydroxy-2,2-dimethyl-6-(2,4,6-trihydroxy-3-methyl-5-acetylbenzyl)-8-cinnamoyl-1,2-chromine)	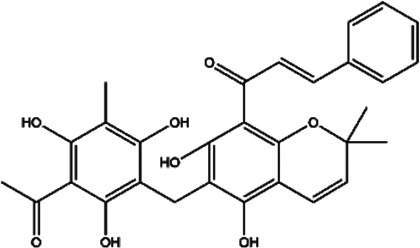	Natural product, PKC δ (protein kinase Cδ) inhibitor	Glioma	Gschwendt et al. (1994)
Compound 34 (Thieno [2–3-b]pyridine analogues)	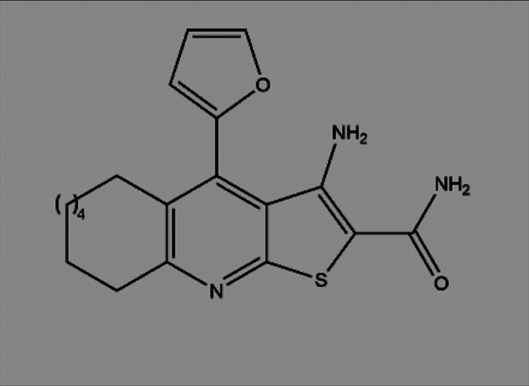	ATP competitive inhibitor	Colorectal cancer	Lockman et al. (2010)
NH125 (1-benzyl-3-cetyl-2-methylimidazolium iodide)	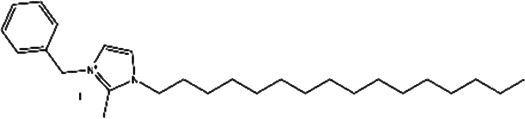	Histidine protein kinase inhibitor, selective inhibitor of eEF2K *in vitro*	Glioma	Arora et al. (2003); Arora et al. (2004); Lockman et al. (2010); Zhang et al. (2011); Zhu et al. (2017); Xie et al. (2018)
			Esophageal squamous cell carcinoma	
			Breast cancer	
			Lung cancer	
Geldanamycin (GA)	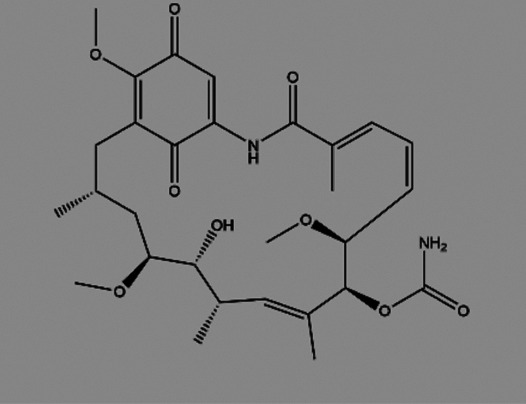	Natural product, blockage of eEF2K and Hsp90	Human glioma	Yang et al. (2001)
17-AAG (17-allylamino-17-demethoxygeldanamycin)	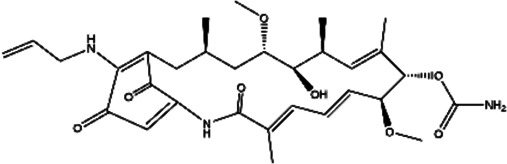	Less toxic and less potent derivative of GA, inhibitor of Hsp/protein interactions	Human glioma	Yang et al. (2001)
Cefatrizine	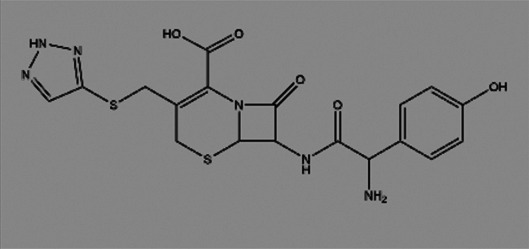	Induces ER stress	Breast cancer	Yao et al. (2016)
Nelfinavir (NFR)	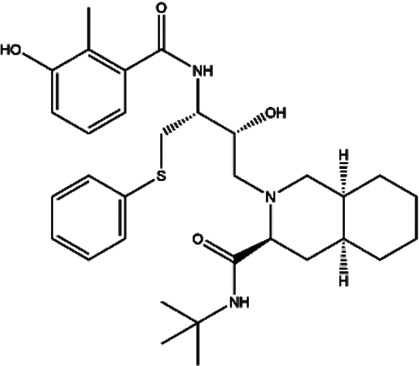	HIV aspartyl protease inhibition, also triggers eEF2K activation	-	De Gassart et al. (2016)
JAN-384, -452, -613, -849	Unknown	Unknown	Breast cancer	Kenney et al. (2016); Moore et al. (2016); Xie et al. (2018)
			Lung cancer	
TS-2 (4-ethyl-4-hydroxy-2-p-tolyl-5,6-dihydro-4H-1,3-selenazine)	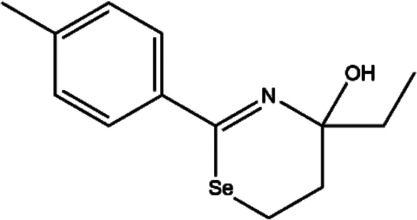	Multiple tyrosine kinase inhibitors, including protein kinase A (PKA), protein kinase C (PKC), and protein tyrosine kinase (PTK)	None	Cho et al. (2000); Lockman et al. (2010)
TS-4 (4-hydroxy-6-isopropyl-4-methyl-2-p-tolyl-5,6-dihydro-4H-1,3-selenazine)	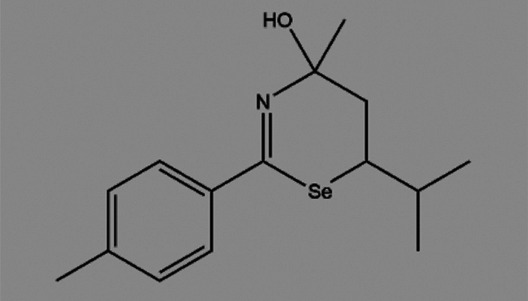	Multiple tyrosine kinase inhibitors, including PKA, PKC, and PTK	None	Cho et al. (2000); Lockman et al. (2010)
TX-1918 (2-hydroxyarylidene-4-cyclopentene-1,3-dione)	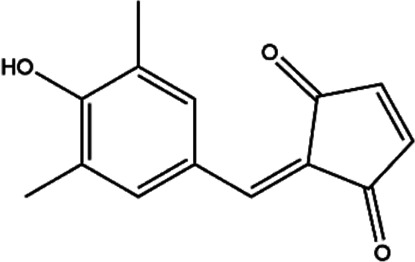	Protein tyrosine kinase inhibitor	TNBC	Hori et al. (2002); Ju et al. (2021)
Compound A1	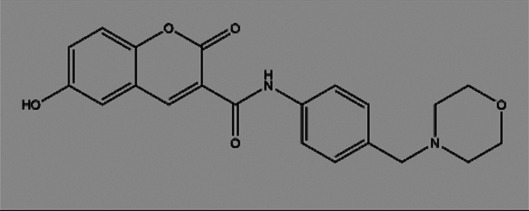	Derived from the core structure of rottlerin; however, the detailed mechanism is unknown	TNBC	Comert Onder et al. (2020)
Compound A2	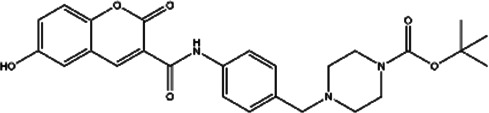	Derived from the core structure of rottlerin; however, the detailed mechanism is unknown	TNBC	Comert Onder et al. (2020)
Thymoquinone (TQ)	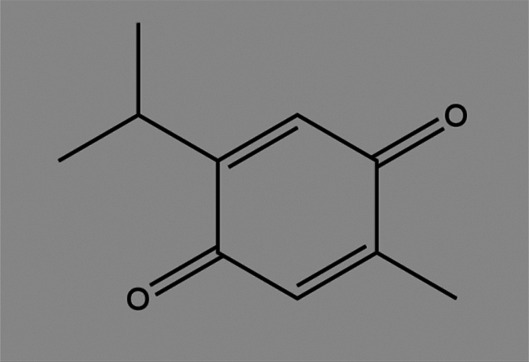	Induction of tumor suppressor, miR-603 and NF-kB inhibition	TNBC	Kabil et al. (2018)
21L (2.4-dichloro)	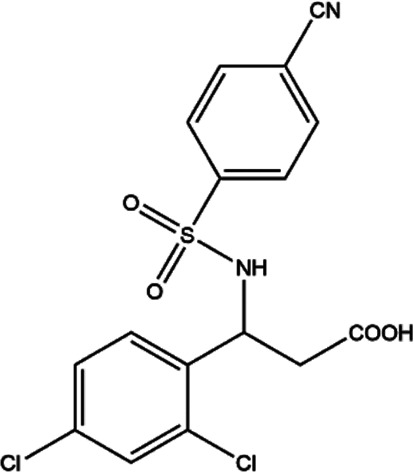	ATP-competitive inhibitor, could stably bind to the ATP binding site of eEF2K	Breast cancer (*in vivo and in vitro*)	Guo et al. (2018)
		Induces apoptosis pathway		

The development of ideal eEF2K inhibitors still faces challenges. Shortcomings regarding eEF2K inhibitors pertain to their lack of selectivity and adverse effects. eEF2K inhibitors are not selective, and this is likely attributed to the lack of the kinase’s crystal structure. Current ATP competitive inhibitors are more efficacious yet cause cytotoxicity in higher concentrations (Liu et al., 2020). Therefore, solving the crystal structure of eEF2K protein and finding proper inhibitors are still in high demand in the future.

Drug combination therapies with eEF2K inhibitors have been used to reduce doses of single drug resistance and increase the efficacy of cancer therapies. Research conducted by Zhu demonstrated the novel idea of combining an eEF2K inhibitor with radiation treatment to treat ESCC (Zhu et al., 2017). Ablation of eEF2K using pharmacological inhibitors with glutamine starvation suppresses TNBC growth (Ju et al., 2021). Silencing eEF2K suppresses the growth and induces apoptosis of breast cancer cells to a drug, doxorubicin (Tekedereli et al., 2012). Combination treatment with an eEF2K inhibitor and an AKT inhibitor (MK-2206) exert a highly anticancer effect on nasopharyngeal carcinoma and glioma (Cheng et al., 2011; Zhao et al., 2018). An anticancer drug, mitoxantrone, a potential inhibitor for eEF2K, can disrupt mTOR inhibitors to enhance the efficacy of anticancer effects in breast cancer cells (Guan et al., 2020).

A novel strategy developed in 2020 targets eEF2K through a route other than inhibition. Proteolysis target chimeric (PROTAC) technology eliminates the eEF2K protein by using a protein hydrolysis mechanism. This new therapeutic mechanism has the advantage of possessing high selectivity, potentially solving the current dilemma of drug resistance. Although future optimization is required in asynchronous pharmacokinetics and pharmacodynamics, the technology is promising (Liu et al., 2020).

## Conclusion

**FIGURE 3:**
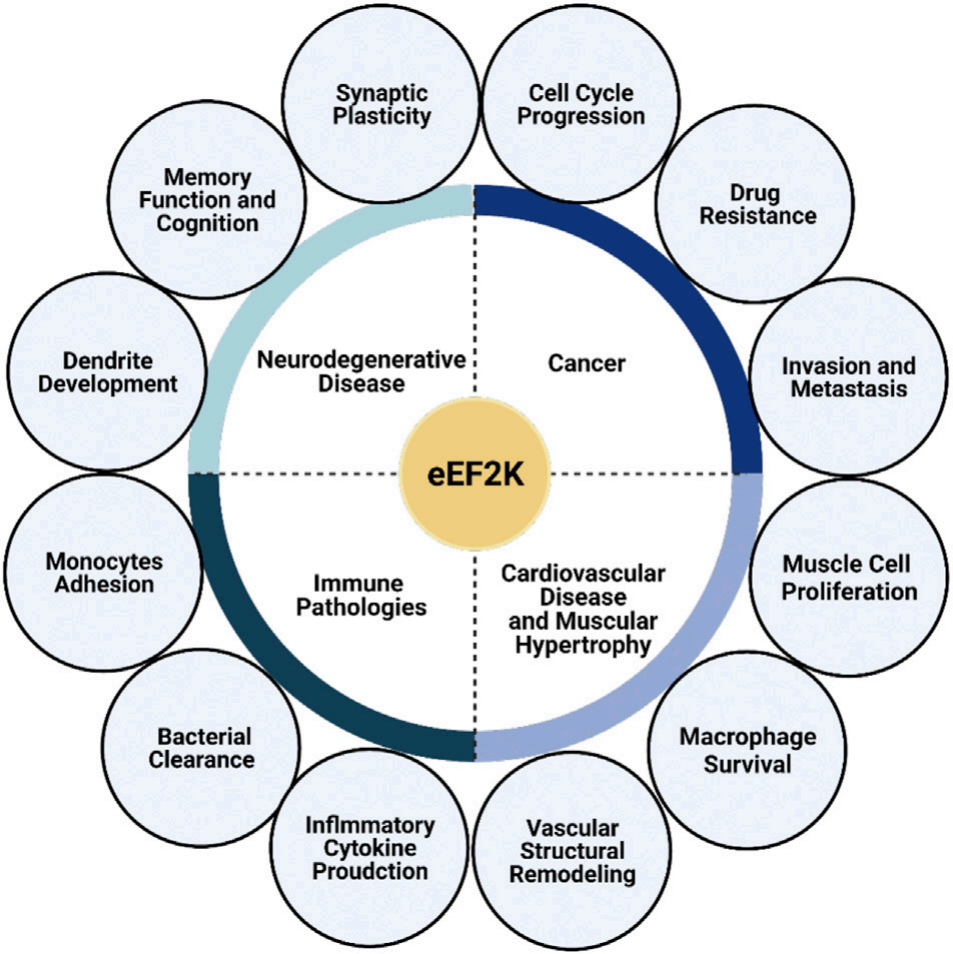
eEF2K plays a significant role in multiple pathologies. eEF2K is expressed at high levels in several diseases, including neurodegenerative diseases, cancer, cardiovascular disease, muscular hypertrophy, and other immune pathologies, causing it to serve as a suitable and promising drug target. eEF2K affects synaptic plasticity, memory function, and dendrite development in neurology. Dysfunction of these traits leads to the development and progression of neurodegenerative diseases. The progression of cancer and cancer-cell growth has also been correlated with eEF2K activity and expression levels.

Protein translation is tightly regulated at the elongation stage of protein synthesis by eEF2K. The mechanisms which regulate eEF2K activity are varied but ultimately controlled by the nutrient status of the cell *via* multiple cell signaling pathways, including AMPK and mTOR. These pathways signal to increase protein synthesis when nutrients are available while decreasing protein synthesis during nutrient deprivation *via* eEF2K phosphorylation of its substrate, eEF2. Metabolic reprogramming *via* the eEF2K pathway is essential to several biological systems, especially in the nervous system and cardiology. Therefore, eEF2K is considered a potential drug target for numerous diseases, including cancers, neurodegenerative diseases, cardiovascular diseases, and other immune pathologies (Figure 3). Moreover, several studies have reported the importance of eEF2K in controlling metabolic processes in immunity both during infection and in autoimmune disorders. Future studies will continue to elucidate the biological significance of this kinase in immunity. Furthermore, targeting eEF2K may help restore normal metabolism in different types of cells under various pathologic conditions.
